# Gastrointestinal symptoms and the Mediterranean dietary pattern: secondary analysis of a randomized controlled trial in a population with increased cardiometabolic risk

**DOI:** 10.3389/fnut.2026.1799576

**Published:** 2026-04-20

**Authors:** Ella Silk, Mark Weatherall, Fiona E. Lithander, Meika Foster, Troy Merry, Andrea Braakhuis, Amber Parry Strong, Cecilia Ross, Denise Conroy, Anna Rolleston, Jeremy Krebs, Richard Gearry

**Affiliations:** 1 Department of Medicine, University of Otago Christchurch, Christchurch, New Zealand; 2 Department of Medicine, University of Otago, Wellington, New Zealand; 3 Liggins Institute, University of Auckland, Auckland, New Zealand; 4 Edible Research, Ohoka, New Zealand; 5 Department of Nutrition, University of Auckland, Grafton Campus, Auckland, New Zealand; 6 Department of Nutrition, University of Auckland, Auckland, New Zealand; 7 Centre for Endocrine, Diabetes and Obesity Research, Te Whatu Ora Health New Zealand Capital, Coast and Hutt Valley, Wellington, New Zealand; 8 Food Innovation, Plant and Food Research, Sandringham, New Zealand; 9 The Centre For Health, Tauranga, New Zealand

**Keywords:** cardiometabolic risk, dietary pattern, gastrointestinal symptoms, Mediterranean diet, quality of life

## Abstract

**Background/objectives:**

Cardiometabolic diseases are a major global health concern, and their development are linked to suboptimal dietary patterns and adverse gastrointestinal (GI) health outcomes and symptoms. Mediterranean dietary patterns are associated with reduced cardiometabolic disease risk and improved quality of life (QoL). The potential impact of the Mediterranean diet on GI symptoms is an important area of inquiry that requires elucidation. This study aimed to evaluate whether a New Zealand-adapted Mediterranean diet (NZMedDiet) affects GI symptom severity in adults at increased cardiometabolic risk, explore the influence of participant characteristics on symptoms, and assess the association between GI symptoms and health-related QoL.

**Methods:**

This secondary analysis used data from a randomized controlled trial of a 12-week NZMedDiet and behavioral intervention in adults with increased cardiometabolic risk. Associations were assessed between the gastrointestinal symptom rating scale (GSRS), the 36-Item Short-Form Survey (SF-36)(assessing QoL), and the NZMedDiet, alongside participant characteristics.

**Results:**

A total of 200 participants were enrolled (mean age 49.9 ± 10.9 years; 62% women), with women reporting significantly higher baseline GI symptom severity scores on average across all GSRS domains except reflux (*p* < 0.05). Māori participants reported higher baseline indigestion and abdominal pain scores compared with New Zealand Europeans (*p* < 0.01). Compared with controls, participants in the NZMedDiet group had lower GSRS domain scores, with the largest between-group estimated mean difference observed for abdominal pain being −0.27 (95% CI: −0.48 to −0.06; *p* = 0.01). Moderate associations were observed between GSRS domains and quality-of-life measures, particularly between abdominal pain and the general health domain (*r_s_* = −0.25; *p* < 0.001).

**Conclusion:**

The NZMedDiet had a positive impact on GI symptom severity scores and was well tolerated in participants with increased cardiometabolic risk. Improvements in GSRS domains were correlated with moderate improvements in QoL measurements.

**Clinical trial registration:**

https://www.anzctr.org.au/Default.aspx, identifier ACTRN12622000906752 and https://www.isrctn.com/, identifier ISRCTN89011056.

## 1 Introduction

Gastrointestinal (GI) symptoms are common even in those who have not been formally diagnosed with a GI disorder. The global prevalence of diagnosed irritable bowel syndrome (IBS) is reported as being between 9 and 11% (1, 2). However, a recent multi-national study reported that over 40% of people globally may have some form of disorder of gut brain interaction (DGBI) (3). This highlights both underdiagnosis and the broader global burden of GI symptoms. Not only are GI symptoms highly prevalent, they can significantly impair quality of life (QoL) in patients with and without a diagnosed DGBI (4).

An important modifiable factor contributing to GI symptoms is diet. Currently, many people consume what is referred to as a ‘Western-style’ diet, which is characterized as energy-dense, low in fiber, and high in foods rich in fat, sugar and salt. People who consume a Western-style diet experience more GI symptoms and have an increased risk of developing a DGBI (5, 6). Modification of the diet may improve or reverse diet-induced GI dysfunction (7).

A dietary pattern that has attracted significant attention is the Mediterranean diet (MedDiet), which is characterized by plant-based foods, legumes, nuts, seafood and dietary fiber with low intakes of saturated fat and red meat and the prominent use of olive oil (8). The MedDiet has been shown to improve cardiometabolic health and has also been positively associated with prevention of GI symptoms (9, 10). The beneficial effects of a MedDiet on GI health may be mediated by alterations to the gut microbiome, because of its role in nutrient uptake and intestinal barrier permeability (11). This association with improved GI symptoms has been observed in the context of patients with IBS and depression (12–14). At present there are no trial data demonstrating the effect of a MedDiet on GI symptoms in those at risk of cardiometabolic disease who do not have a diagnosed GI disorder. Consequently, available evidence is largely derived from populations with conditions such as IBS, which represents the closest available evidence base. Given the high prevalence and underdiagnosis of DGBI, GI symptoms remain relevant in this population and may be influenced by dietary change. Demonstrating that the MedDiet is well tolerated in this population would support its broader implementation, given the established benefits for cardiometabolic health and longevity (15–17).

While a MedDiet is reported to generally improve GI symptoms, some evidence suggests that certain foods may exacerbate symptoms, for example, fruits and vegetables have been associated with increased IBS symptom severity score (18). While otherwise healthy individuals with IBS differ from those with cardiometabolic health risks, it remains important to ensure that the MedDiet does not trigger GI symptoms in those at risk of cardiometabolic disease. If GI function improves or remains unchanged, this supports recommending a MedDiet as a long-term dietary strategy for individuals with underlying cardiometabolic risk.

This study focuses on the effects of consuming a New Zealand-adapted MedDiet (NZMedDiet) on GI symptoms, and their association with QoL, in a population at risk of cardiometabolic disease, as part of a larger multi-center prospective randomized controlled trial (RCT), *He Rourou Whai Painga* (HRWP) (19). Although participants were not selected based on experiencing GI symptoms, GI symptom outcomes were included as secondary outcomes given the high prevalence of such symptoms in the general population (4), and the likelihood that substantial changes to dietary intake could affect GI function. This allowed assessment of whether the intervention improved, had no effect, or exacerbated GI symptoms, to support the feasibility of recommending the NZMedDiet to individuals at cardiometabolic risk regardless of GI symptom status. This trial is among the few designed to investigate the impact of a MedDiet on the health of New Zealand adults, and the first to explore its potential to improve GI health in relation to QoL. This secondary analysis therefore assessed changes in GI symptom severity following the NZMedDiet intervention, explored associations between participant characteristics and GI symptoms, and assessed whether changes in GI symptom severity were related to QoL in adults at risk of cardiometabolic disease. While HRWP has previously reported on primary cardiometabolic and QoL outcomes (20), the relationship between GI symptom severity, participant demographics and QoL outcomes were not examined.

## 2 Materials and methods

### 2.1 Trial design and participants

The full HRWP trial design, setting and eligibility criteria have been published elsewhere (19). In short, HRWP was a multi-center, multi-stage trial conducted across four centers in New Zealand, involving 200 index participants and their households, including a 12-week NZMedDiet intervention phase. For this analysis, only RCT1 of HRWP was utilized. In RCT1, one group completed a 12-week habitual diet phase before commencing the 12-week NZMedDiet intervention, while the other began the intervention immediately after randomization; both groups therefore completed the NZMedDiet intervention phase. Over the 12-week dietary intervention phase, HRWP participants were provided with food and beverages comprising approximately 75% of their daily energy requirements, delivered to participants’ homes free of charge, with the remainder sourced by participants. The foods and beverages provided, which are outlined in detail in a prior publication (21), were high-quality New Zealand foods, emphasizing fruits, vegetables, grains, legumes, seafood, nuts, and some meat and dairy. During the intervention phase, participants were also provided with online recipes, web-based education, and opt-in online social support, to encourage adherence to the NZMedDiet. HRWP participants were recruited based on having a high risk of developing cardiometabolic disease, using a metabolic syndrome severity score (MetSSS) > 0.35 (20, 22). Further inclusion and exclusion criteria have been detailed previously (19); of relevance to the present analysis, the exclusion criteria included ‘disorders that impact digestion or nutrient absorption, as well as previous bariatric surgery’.

### 2.2 Data collection

**Figure 1:**
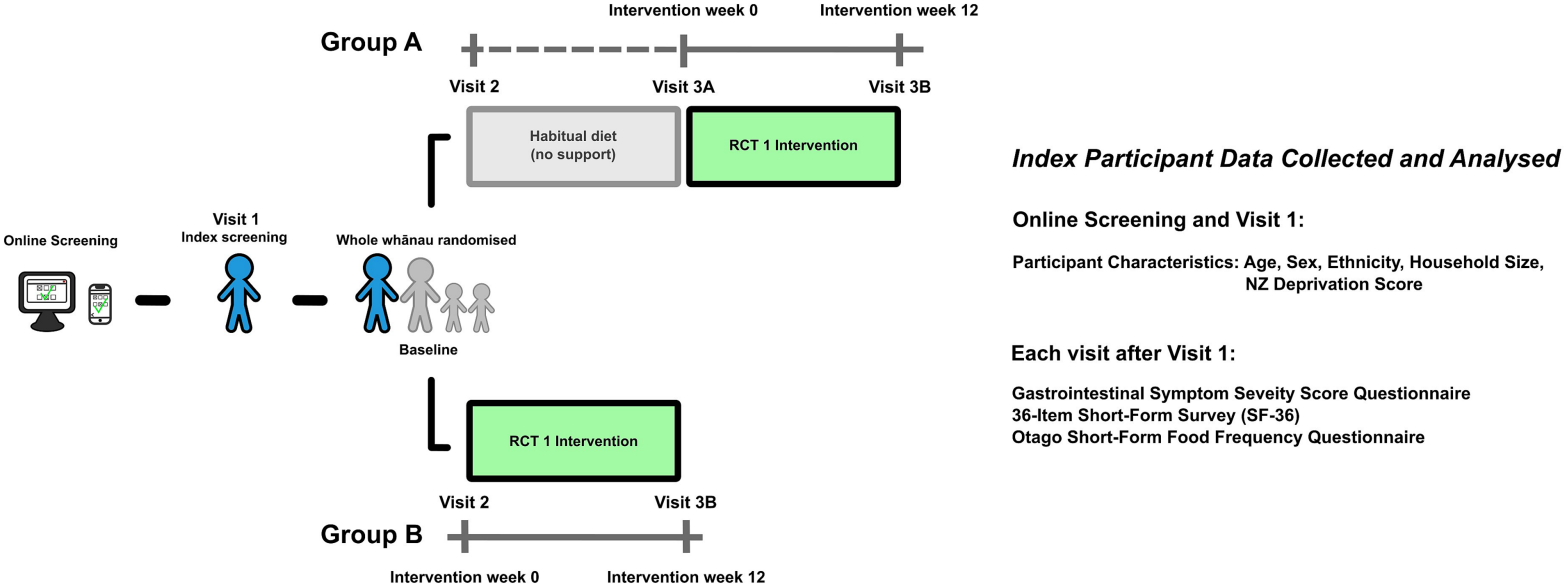
HRWP study design excerpt showing time points used for analysis and measurements of interest. As part of the HRWP multi-stage trial design, participants were randomized to two groups, A and B. Group A participants consumed their habitual diet for 12 weeks before commencing the 12 week dietary intervention. Group B began the dietary intervention immediately after randomization.

HRWP participant demographic data (age, sex, ethnicity and household size) were collected at the screening visit, while NZ Socioeconomic Deprivation index (NZDep), an area-based measure of socioeconomic deprivation, was measured using participant household addresses. Questionnaires used in this analysis, the Gastrointestinal Symptom Rating Scale (GSRS), 36-Item Short-Form Survey (SF-36) and Otago short-form food frequency questionnaire (FFQ), were administered at every visit after screening (Control Group A: visit 2, visit 3a and visit 3b; NZMedDiet Group B: visit 2, visit 3b; Figure 1), electronically or on paper, as preferred.

### 2.3 Questionnaires

The GSRS (23) was used to examine the potential impact of the NZMedDiet on GI symptoms. The GSRS tool assesses GI symptoms by combining 15 items into five symptom clusters, namely, abdominal pain, reflux syndrome, diarrhea syndrome, indigestion syndrome and constipation. The higher the score ascribed to each cluster, the more severe the symptoms.

The SF-36 (24) was used to assess health-related QoL. This tool measures self-reported health using a scoring key, which covers 8 domains: Physical activity limitations because of health, social activity limitations because of physical or emotional issues, role limitations due to physical health issues, role limitations due to emotional issues, bodily pain, general mental health, vitality (fatigue and energy levels) and perceptions of general health. The component summary scores are the Physical Component Summary (PCS) for physical-health related elements, and the Mental Component Summary (MCS) for mental-health related elements.

The extent to which the achieved diet was related to a MedDiet was assessed by the Otago short-form FFQ (25): The instrument was used to calculate the Pyramid based Mediterranean Diet Score (PyrMDS), a 15-component score measuring how ‘Mediterranean’ the achieved diet was. The PyrMDS has been shown to have fewer flaws and a strong supporting body of theoretical and scientific evidence than alternative instruments (26).

### 2.4 Sample size and statistical analysis

The sample size for the HRWP trial was based on detecting an important difference in the MetSSS, a Metabolic Risk score.

Analysis of covariance (ANCOVA) models were used to estimate adjusted mean GSRS scores comparing the control groups habitual diet phase to NZMedDiet groups intervention phase, adjusting for baseline values (visit 2). All other analyses utilized the intervention phase for both groups, with intervention week 0 as baseline and intervention week 12 as the post-intervention time point.

Associations between GI symptoms scores and participant characteristics at intervention week 0 for categorical variables were determined using one-way analysis of variance (ANOVA), in which the square root of *R*-squared value (√*R*^2^) is reported to provide an effect size that was equivalent to a Pearson’s correlation coefficient. Mann–Whitney tests with Hodges–Lehmann estimates of median differences were used for dichotomous categorical comparisons. Kruskal–Wallis tests were used for comparisons across multiple ethnic groups. Associations between GI symptoms and continuous participant characteristics were explored using Spearman’s rank-based correlation coefficient (*r_s_*) and regression coefficient estimation (B), which includes change in PyrMDS score over the intervention period. The *p*-values for the regression coefficients were equivalent to those derived from Pearson’s product–moment correlation coefficient.

Associations between change in SF-36 dimensions and GSRS scores were determined using Spearman’s rank-based correlation coefficient and regression coefficient estimation.

A *p*-value < 0.05 was considered statistically significant.

SAS version 9.4 (SAS Institute Inc., Cary, NC, USA) and GraphPad Prism version 10.5.0 were used for analysis.

### 2.5 Ethics statement

All participants provided written informed consent prior to participation. Ethical approval was granted by the New Zealand Health and Disability Ethics Committee (Northern B Committee) (reference 2022 FULL 12045).

## 3 Results

### 3.1 Participant demographics

Participant characteristics for the 200 participants included in this analysis are shown in Table 1. The cohort was predominantly NZ European (49%), followed by Māori (29%) and Pacific Islander (14%). Females comprised 62% of the population. The mean (SD) age was 49.9 (10.9) y, ranging from 23.4 to 68.5 y. The mean and median socioeconomic status of the participants at baseline (*N* = 198) was 5 (SD = 2.7) and 5 (IQR = 2–7), respectively (data not shown). This is consistent with a mid-range level of deprivation, with moderate variability. When household size was categorized, 48% of households included two family members, including the index participant, and 52% had more than two family members.

**Table 1:** HRWP Participant characteristics.

Category	*N*/200 (%)
Ethnicity	
Asian	15 (8)
European	98 (49)
Māori	57 (29)
MELAA	1 (0.5)
Other	2 (1)
Pacific Islander	27 (14)
Sex (Female)	124 (62)

Household size	*N*/199 (%)
2	96 (48)
3	35 (17)
4	23 (12)
5	21 (16)
6	6 (3)
7	3 (2)
8	5 (3)
Household size greater than 2	103 (52)

MELAA, Middle Eastern, Latin American, African.

### 3.2 Adjusted between-group differences in GSRS domain scores at week 12 (NZMedDiet vs. control)

Firstly, ANCOVAs were conducted for each GSRS domain at week 12 for both the control and NZMedDiet group, adjusting for baseline GSRS scores (Table 2). All GSRS domains displayed small but statistically significant adjusted mean differences favoring the intervention group, with abdominal pain having the largest estimated mean difference (CI) being −0.27 (−0.48 to −0.06, *p* = 0.01). These findings were used to inform subsequent analyses conducted during the intervention period for all participants. Supplementary Table 1 displays specific changes in scores between groups.

**Table 2:** Estimated GSRS domain score differences in control and intervention at 12 weeks.

GSRS variable	Week 12; diet minus control estimate (95% CI)^a^	*p*
Abdominal pain	−0.27 (−0.48 to −0.06)	0.01
Constipation	−0.22 (−0.44 to −0.004)	0.046
Diarrhea	−0.18 (−0.39 to 0.04)	0.011
Indigestion	−0.21 (−0.41 to −0.02)	0.034
Reflux	−0.26 (−0.50 to −0.02)	0.037

a Baseline GSRS domain scores used as a continuous covariate.

### 3.3 Association between demographic variables and GSRS component scores

Despite not reporting diagnosed GI disorders at screening, women were more likely than men to report more severe GI symptoms in all sub-components of the GSRS (*p* < 0.05) except for reflux. The greatest baseline gender difference was observed for abdominal pain, where there was a difference in means (95% CI) of −0.43 (−0.66 to −0.2, *p* < 0.001) (Supplementary Tables 2 and 3). Ethnicities other than NZ European were associated with a significantly increased risk of abdominal pain (√*R*^2^ = 0.26, *p* = 0.004) and indigestion compared with NZ European (√*R*^2^ = 0.23, *p* = 0.02) (Supplementary Table 4). Specifically, Māori participants had significantly worse abdominal pain and indigestion compared to NZ Europeans, with a difference in means (95% CI) of 0.47 (0.2–0.73, *p* < 0.001) and 0.4 (0.14–0.66, *p* = 0.003), respectively (Supplementary Table 5). A weak negative association was observed between NZDep and abdominal pain at baseline (*r_s_* = −0.15, *p* = 0.035), however, this relationship did not remain significant when modelled using regression estimates (Supplementary Table 6). There were no significant associations between baseline GSRS component scores and age, household size or baseline adherence to the NZMedDiet (PyrMDS) (Supplementary Tables 7–10). Similarly, no associations were observed between changes in GSRS domain scores and changes in PyrMDS total score over the intervention period (Supplementary Table 11).

### 3.4 Quality of life is associated with improved gut symptoms post-intervention

**Figure 2:**
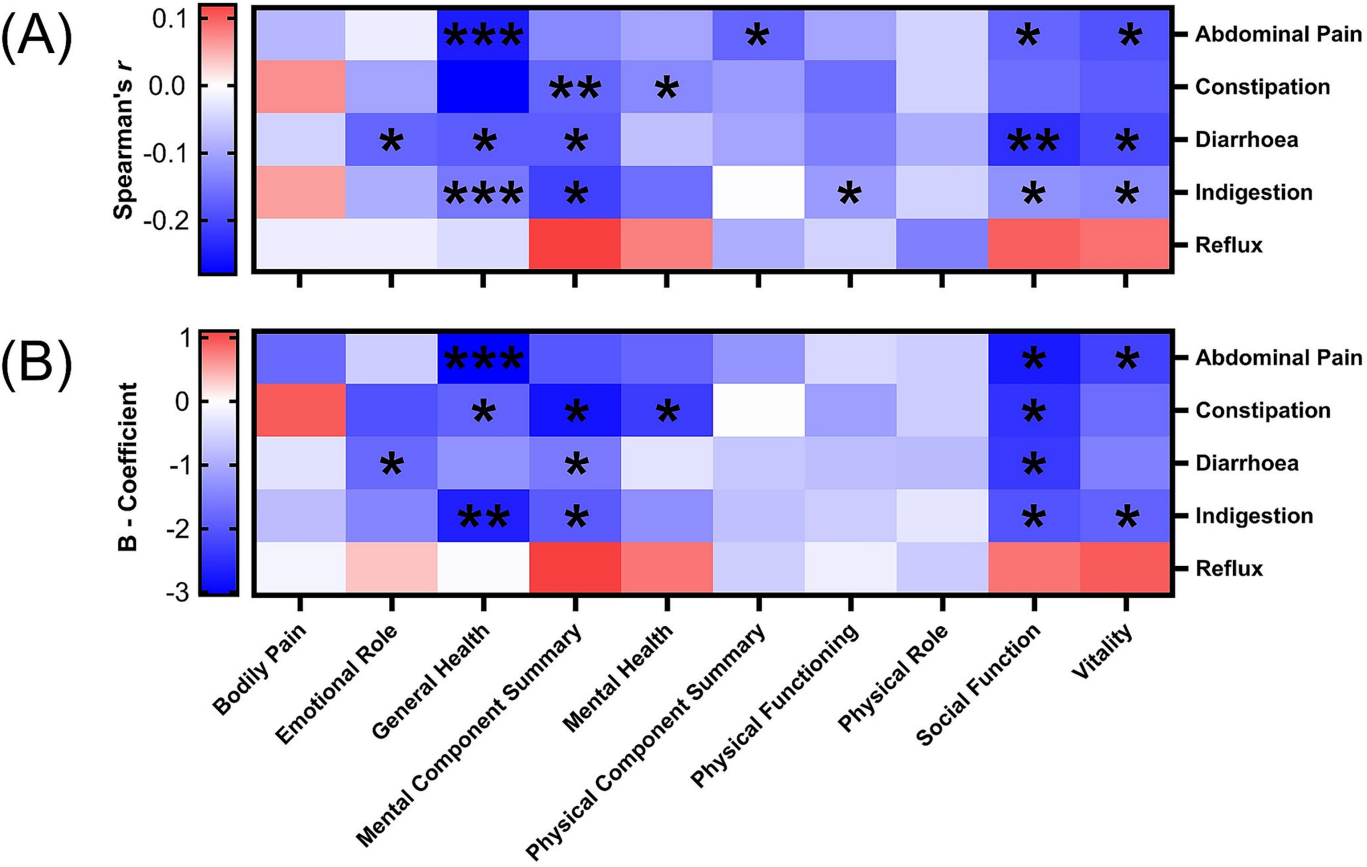
Correlation and regression estimates for the relationship between SF-36 dimension scores and GSRS domains. **(A)** Spearman’s rho correlations. **(B)** B-coefficients represent the change in SF-36 dimension per unit increase in GSRS domain score. Specific statistical findings are located in Supplementary Tables 12 and 13. Significance is represented by ^*^p < 0.05, ^**^p < 0.01 and ^***^p < 0.001.

Previously published analysis showed significant improvements in multiple quality of life components of the SF36 in the intervention group compared to the control group (20). Improvements in abdominal pain, constipation, diarrhea, and indigestion were associated with improvements in at least three of the SF-36 dimensions, particularly general health, physical component summary, social function and vitality. Reflux was not significantly associated with change in any of the SF-36 components. Supplementary Tables 12 and 13 demonstrate specific correlation and regression analyses between SF-36 domains and GSRS domain scores, which are presented in Figure 2.

Firstly, general health was negatively associated with several GSRS domains and displayed the strongest associations with abdominal pain (*r_s_* = −0.25, *B* = −3.05) and indigestion (*r_s_* = −0.28, *B* = −2.68) (all *p* < 0.001). Significant but weaker associations were also observed with constipation (*B* = −1.88, *p* = 0.03) and diarrhea (*r_s_* = −0.18, *p* = 0.02).

The MCS score was weakly correlated with constipation (*r_s_* = −0.21, *p* = 0.006; *B* = −2.83, *p* = 0.01) and indigestion (*r_s_* = −0.17, *p* = 0.02; *B* = −1.98, *p* = 0.03). The mental health score further supported the association with constipation (*r_s_* = −0.16, *p* = 0.04; *B* = −2.34, *p* = 0.02). In contrast, the PCS score displayed one weak correlation with abdominal pain (*r_s_* = −0.17, *p* = 0.03), which was not significant in regression. Physical role and bodily pain were not significantly associated with any GSRS domain.

While having weaker significance, social function had associations across all domains bar reflux, however was only significant across both testing methods for abdominal pain (*r_s_* = −0.19, *p* = 0.03; *B* = −2.75, *p* = 0.01), diarrhea (*r_s_* = −0.23, *p* = 0.003; *B* = −2.37, *p* = 0.01) and indigestion (*r_s_* = −0.18, *p* = 0.02; *B* = −2.06, *p* = 0.04). Vitality was consistently related with abdominal pain (*r_s_* = −0.19, *p* = 0.02; *B* = −2.28, *p* = 0.02) and indigestion (*r_s_* = −0.18, *p* = 0.02; *B* = −1.88, *p* = 0.04).

## 4 Discussion

This study aimed to assess how NZMedDiet consumption, participant demographics, and QoL, impact GI symptom severity. The NZMedDiet was associated with minor, but statistically significant, decreases in all GSRS domain scores. This is an important observation, as it affirms another positive health benefit that a Mediterranean-style diet is reported to have in patients that are at risk of developing cardiometabolic disease. A second major finding from this analysis was the existence of a clear relationship between improved GSRS domain scores and enhanced QoL, as indicated by the multiple positive associations between decreased GSRS domain scores and changes in SF-36 outcomes after consumption of the NZMedDiet.

### 4.1 The NZMedDiet is associated with decreases in gastrointestinal symptom severity score

The NZMedDiet was associated with modest decreases in GSRS domain scores; when accounting for baseline values, participants in the intervention group reported significantly lower domain scores at week 12 compared to controls. Although prior studies in populations with higher baseline GI symptom burden, such as those with IBS, Parkinson’s disease, or older adults, often used different instruments and observed larger associations and absolute reductions in symptoms, the modest decreases seen here are consistent with these findings and are expected given the lower baseline symptom severity in our cohort (12, 14, 27). The findings presented here provide preliminary evidence that the MedDiet also supports GI comfort in a population with low-grade or sub-clinical GI symptoms, supporting the use of a MedDiet to improve overall wellbeing.

### 4.2 Extrapolation of findings to the general population

Having periodic GI symptoms is common in the general population, particularly in women, even in the absence of diagnosed GI disorders. This phenomenon has been well reported in the literature (3, 28), and clinical studies consistently show women are more likely than men to report bloating, abdominal pain and other pain-related IBS diagnostic symptoms (29–31). Consistent with these reported characteristics, the participants in this analysis reported GI symptoms despite not having diagnosed GI disorders, with female participants reporting significantly worse gut symptoms, particularly abdominal pain, compared to males. This relationship is thought to be due to differences in stress, sex hormones and menstruation (28).

There is also some evidence to suggest that sub-diagnostic GI symptom prevalence and severity differs by ethnicity (32, 33). In this analysis, ethnicity was shown to have a significant association with symptom severity. Specifically, Māori had worsened severity in both abdominal pain and indigestion domains and appeared to drive the significance found by ethnicity overall, which aligns with broader health inequities affecting Māori, including those related to upper GI health (34). For example, Māori experience more *Helicobacter pylori* infections, increasing the risk of developing peptic ulcers, which can cause multiple GI symptoms (35).

Differences in GI symptoms by ethnicity are likely influenced by a complex interplay of genetic, environmental, and socioeconomic factors, including diet. The typical New Zealand diet is characterized by energy-dense, foods rich in fat, sugar and salt, saturated fat and red meat (36), reflecting a Western-style dietary pattern. Māori adults tend to have higher absolute energy intakes and larger portion sizes compared to NZ Europeans, reflecting a greater consumption of fat and carbohydrate (36), which may somewhat explain the reporting of more severe GI symptoms in Māori. Previous literature on food intake behavior and fat intake (37, 38) suggests that diets rich in fat can increase dyspeptic symptoms and bloating, while greater energy intake required to reach satiety has been associated with higher risk of abdominal pain. These factors may partially explain the reporting of more severe GI symptoms in Māori. Increasing evidence (5, 6, 39) suggests negative associations between intake of a Western dietary pattern and overall wellbeing highlights the critical importance of research that identifies how diets can be changed to improve health outcomes in the New Zealand population and across ethnicities.

### 4.3 Associations between gastrointestinal symptoms and quality of life

In general, it is known that QoL is positively associated with GI health and negatively associated with symptom prevalence and severity (4, 13, 40). In this analysis, numerous significant inverse relationships were observed between SF-36 outcomes, as a measure of QoL, and all of the GSRS domains except reflux, affirming the positive impact of the MedDiet on mental and physical health. General health had the most, and the strongest associations, which were abdominal pain, constipation, diarrhea, and indigestion. This suggests that improvements in GSRS domain scores are influencing the overall positive feeling of health within the cohort.

This observed relationship between gut comfort and general health accords with other evidence on MedDiet intake and adherence to this dietary pattern (12, 14), which has been associated with improved GI symptoms in IBS, but also with reductions in anxiety, depression and overall IBS-QoL score (12). Similarly, in this analysis, the MCS score indicated a relationship between improvements in constipation and indigestion with improved mental health. In contrast, the PCS score demonstrated minimal associations with GSRS domains, suggesting that GI symptoms in this cohort exerted a greater influence on perceived mental and general wellbeing than on physical functioning. Furthermore, similar patterns have also been observed in an ageing population, where consumption of a 3 month MedDiet led to significant increases in general health (14). Importantly, these associations are not exclusive to adults. In adolescents with physical or developmental disabilities (a population known to experience problematic GI function), it was observed that having a higher MedDiet score was associated with lower total GSRS and domain scores, and fewer nutrient deficiencies (40). Furthermore, increases in GSRS scores were linked to a reduction in pediatric QoL score (40), which reinforces the importance of diet quality, GI health and QoL at all ages.

While causation cannot be established from this dataset, the observations are consistent with what is known regarding the bidirectional communication of the gut-brain axis. The MedDiet is rich in anti-inflammatory (e.g., unsaturated fats) and antioxidant compounds and phenols from fruits, vegetables and olive oil that can neutralize free radicals, reducing cellular damage and inflammation in the gut (41–43). It is likely that these compounds together with other features of the dietary pattern, such as high fiber, act synergistically to promote gut health and, in turn, brain health (44). Furthermore, the MedDiet is thought to influence the gut-brain axis through promotion of beneficial microbes and their associated metabolites (44, 45). One major example of this is bacterial taxa that can utilize dietary fiber and resistant starch (saccharolytic bacteria) to produce short chain fatty acids (SCFA), which have known roles in maintaining gut barrier function and immune regulation (45). SFCAs have been implicated in GI conditions such as IBS, where lower levels are observed in those with constipation-predominant IBS (46, 47). Increased SCFA production through MedDiet-associated fiber fermentation may contribute to the decreases in constipation and abdominal pain scores observed in this cohort (48, 49). Similarly, the reduction in indigestion and diarrhea symptom scores may reflect reduced gut inflammation driven by bioactive compounds within the diet (41–43). Through this gut-microbiome-brain interface, GI symptom improvements through modification of the diet are likely linked with enhanced QoL (50). Whether the observed reduction in GSRS scores in this analysis are attributable to individual dietary components or their synergistic combination remains to be determined, and represents an important area for future investigation.

### 4.4 Minimum clinically important differences in GSRS

While significant changes in GSRS domains between control and intervention groups were observed, with the largest estimated mean difference being 0.27 for abdominal pain, it is important to acknowledge that these shifts may not reach thresholds typically considered to be clinically meaningful. While it has been determined that GSRS is a valid anchor to detect patient-reported outcomes (51), no minimum clinically important differences (MCID) have been established for patients with elevated cardiometabolic risk, such as the HRWP study population.

MCIDs have been reported in other clinical contexts. For example, a GSRS diarrhea MCID of 0.33 was determined in patients with Fabry disease (52). In renal transplant patients, MCIDs were estimated for all GSRS domains: 0.6 for abdominal pain, 0.8 for reflux, 0.4 for diarrhea, 0.7 for indigestion and 0.7 for constipation (53). The effects observed in this study were modest compared to these MCIDs, which likely reflects the lower baseline symptom burden in this cohort. These populations differ greatly from the HRWP population in disease burden and symptom presentation, which may limit the applicability of these scores to our findings, highlighting the need to better understand MCID for GSRS scores in cardiometabolic and dietary intervention populations.

### 4.5 Limitations

This study provides novel insights into the effects of a MedDiet on GI symptoms in a New Zealand population at risk of cardiometabolic disease; however, several limitations must be acknowledged. Firstly, due to the cross-sectional nature of much of the analyses undertaken on this dataset, causality cannot be determined. Because the initial analysis compared week 12 outcomes while adjusting for baseline values, the results represent adjusted between-group differences rather than true longitudinal changes. Consequently, some individual-level intervention effects may not have been fully captured.

As the analysis reported here is a secondary analysis, there may be type II errors for associations that were not statistically significant, particularly in the analysis involving ethnicities was limited by variation in sample size between groups. Further, these analyses are not adjusted for multiplicity so type I error inflation may be present for statistically significant associations, and marginal associations should be taken with caution. Given that GSRS scores were generally low in this analysis, floor effects may have limited the estimated mean differences between control and diet groups post-intervention.

Additionally, the GSRS questionnaire has not been formally validated in a NZ population, therefore it cannot be assumed that all NZ ethnic groups interpreted or responded to the survey in a comparable way, leading to the possibility of an ethnic response bias. In this analysis, dietary intake was assessed using PyrMDS scores, derived from FFQ data. While the FFQ is an accepted tool for capturing changes in dietary intake, it can be limited by recall bias, fixed food options, and may have reduced sensitivity for detecting dietary changes compared to 24-h food recalls (54). Finally, as ~75% of the participants’ dietary intake was provided during the intervention, the initial RCT1 results may not reflect the food choices participants would otherwise have made, nor be extrapolatable to the general population.

### 4.6 Future directions

In future studies, it would be useful to assess differences in GI symptom prevalence and severity between ethnic groups within NZ, as there is currently a lack of information in this area. In conjunction, validation of GSRS in the NZ context would allow reliable interpretation across the diverse NZ population. Determining GSRS MCIDs for general populations would be beneficial for future studies to determine if certain dietary interventions have important roles in maintaining or improving gut function. Further, examining the potential interrelationships between gut comfort, QoL and the gut microbiome in the population under study could support the possible mediatory role of the gut-brain axis in improving GSRS and SF-36 scores.

## 5 Conclusion

In conclusion, the NZMedDiet was associated with favorable shifts in GI symptom distributions in individuals with elevated cardiometabolic risk, however, it is currently unclear whether the magnitude of the changes would be clinically meaningful. Baseline characteristics were representative of the general population, with females having higher GSRS domain scores compared to males, and Māori having higher scores in both abdominal pain and indigestion compared to NZ Europeans. Finally, physical, mental and social QoL categories were inversely associated with GSRS domains, supporting the potential role of diet in mediating the gut-brain axis and general wellbeing.

## Data Availability

The raw data supporting the conclusions of this article will be made available by the authors, without undue reservation.
