# Supplementary material for: Gastrointestinal symptoms and the Mediterranean dietary pattern: secondary analysis of a randomized controlled trial in a population with increased cardiometabolic risk
Source: Front Nutr. 2026 Apr 20;13:1799576. doi: 10.3389/fnut.2026.1799576 (PMC13136008; doi:10.3389/fnut.2026.1799576)
Supplement: Supplementary file 1 [file Table_1.DOCX]

Supplementary Material

# Supplementary Data

# Supplementary Figures and Tables

Table 1. Baseline and Week12 in RCT1 GSRS scores and change from baseline for each randomized group

| **GSRS variables** |  |  |  |
| --- | --- | --- | --- |
| **Control** | **Mean (SD)** | **Median (IQR)** | **Min to Max** |
| Abdominal Pain Week 12 N=93 | 1.72 (0.91) | 1.33 (1 to 2) | 1 to 5 |
| Abdominal Pain Baseline N=95 | 1.73 (0.68) | 1.67 (1.33 to 2) | 1 to 3.7 |
| Abdominal Pain Difference N=93 | -0.02 (0.89) | 0 (-0.33 to 0.33) | -2.7 to 3 |
| Constipation Week 12 N=93 | 1.78 (0.99) | 1.33 (1 to 2) | 1 to 5.7 |
| Constipation Baseline N=95 | 1.64 (0.8) | 1.33 (1 to 2) | 1 to 4.3 |
| Constipation Difference N=93 | 0.15 (0.84) | 0 (-0.33 to 0.33) | -2.7 to 3 |
| Diarrhoea Week 12 N=93 | 1.73 (1.04) | 1.33 (1 to 2) | 1 to 6.3 |
| Diarrhoea Baseline N=94 | 1.91 (1.09) | 1.5 (1 to 2.67) | 1 to 5.3 |
| Diarrhoea Difference N=92 | -0.2 (0.96) | 0 (-0.67 to 0.33) | -3 to 3.3 |
| Indigestion Week 12 N=93 | 2 (0.87) | 1.75 (1.25 to 2.5) | 1 to 5 |
| Indigestion Baseline N=95 | 2.08 (0.9) | 1.75 (1.5 to 2.5) | 1 to 4.8 |
| Indigestion Difference N=93 | -0.09 (0.84) | 0 (-0.5 to 0.25) | -2.8 to 2.3 |
| Reflux Week 12 N=93 | 1.68 (1.12) | 1 (1 to 2) | 1 to 6 |
| Reflux Baseline N=95 | 1.64 (1.06) | 1 (1 to 2) | 1 to 6 |
| Reflux Difference N=93 | 0.04 (1.11) | 0 (-0.5 to 0.5) | -5 to 4 |
| **Diet/behavioral intervention** | **Mean (SD)** | **Median (IQR)** | **Min to Max** |
| Abdominal Pain Week 12 N=92 | 1.38 (0.52) | 1.33 (1 to 1.33) | 1 to 3.3 |
| Abdominal Pain Baseline N=99 | 1.6 (0.66) | 1.33 (1 to 2) | 1 to 4.3 |
| Abdominal Pain Difference N=91 | -0.17 (0.72) | 0 (-0.67 to 0) | -2.7 to 2.3 |
| Constipation Week 12 N=92 | 1.5 (0.94) | 1 (1 to 1.67) | 1 to 6.3 |
| Constipation Baseline N=99 | 1.61 (0.87) | 1.33 (1 to 1.67) | 1 to 5 |
| Constipation Difference N=91 | -0.05 (0.71) | 0 (-0.33 to 0) | -1.7 to 2.7 |
| Diarrhoea Week 12 N=92 | 1.34 (0.58) | 1 (1 to 1.33) | 1 to 3.7 |
| Diarrhoea Baseline N=99 | 1.53 (0.77) | 1.33 (1 to 1.67) | 1 to 5.7 |
| Diarrhoea Difference N=91 | -0.15 (0.73) | 0 (-0.33 to 0) | -3 to 2 |
| Indigestion Week 12 N=92 | 1.67 (0.63) | 1.5 (1.25 to 2) | 1 to 3.5 |
| Indigestion Baseline N=99 | 1.86 (0.7) | 1.75 (1.25 to 2.5) | 1 to 3.8 |
| Indigestion Difference N=91 | -0.17 (0.78) | 0 (-0.75 to 0.5) | -2.8 to 1.8 |
| Reflux Week 12 N=92 | 1.34 (0.78) | 1 (1 to 1.25) | 1 to 4.5 |
| Reflux Baseline N=99 | 1.47 (0.82) | 1 (1 to 1.5) | 1 to 5 |
| Reflux Difference N=91 | -0.14 (0.74) | 0 (-0.5 to 0) | -2.5 to 3.5 |

Table 2. ANOVA for associations between GSRS domains and sex at intervention week 0

|  | **Male minus female Estimate (95% CI)** | **R-squared square root (**√R²**)** | **P** |
| --- | --- | --- | --- |
| Abdominal pain | -0.43 (-0.66 to -0.21) | 0.27 | **<0.001** |
| Constipation | -0.35 (-0.62 to 0.08) | 0.18 | **0.01** |
| Diarrhoea | -0.34 (-0.61 to -0.08) | 0.18 | **0.01** |
| Indigestion | -0.28 (-0.51 to -0.05) | 0.17 | **0.02** |
| Reflux | 0.02 (-0.27 to 0.31) | 0.01 | 0.88 |

Table 3. Mann-Whitney test for associations between GSRS domains and sex at intervention week 0

|  | **Male minus Female**  **Hodges-Lehmann estimate (95% CI)** | **P** |
| --- | --- | --- |
| Abdominal pain | -0.33 (-0.33 to -0.33) | **<0.001** |
| Constipation | -0.17 (-0.33 to 0) | **0.01** |
| Diarrhoea | -0.17 (-0.33 to 0) | **0.03** |
| Indigestion | -0.25 (-0.5 to 0) | **0.02** |
| Reflux | 0 (0 to 0) | 0.94 |

Table 4. ANOVA for associations between GSRS domains and ethnicity at intervention week 0

| **Abdominal pain** | **Ethnicity minus European (95% CI)** | **R-squared square root (**√R²**)** | **P** |
| --- | --- | --- | --- |
| Overall effect |  | 0.26 | **0.004** |
| Asian | -0.06 (-0.48 to 0.36) |  | 0.78 |
| Māori | 0.47 (0.2 to 0.73) |  | **<0.001** |
| Pacific | 0.05 (-0.29 to 0.38) |  | 0.77 |
| **Constipation** |  |  |  |
| Overall effect |  | 0.15 | 0.27 |
| Asian | 0.17 (-0.33 to 0.68) |  | 0.49 |
| Māori | 0.29 (-0.02 to 0.6) |  | 0.06 |
| Pacific | 0.24 (-0.16 to 0.63) |  | 0.24 |
| **Diarrhoea** |  |  |  |
| Overall effect |  | 0.07 | 0.83 |
| Asian | -0.16 (-0.65 to 0.33) |  | 0.52 |
| Māori | 0.08 (-0.22 to 0.38) |  | 0.59 |
| Pacific | 0.01 (-0.38 to 0.4) |  | 0.95 |
| **Indigestion** |  |  |  |
| Overall effect |  | 0.23 | **0.02** |
| Asian | -0.07 (-0.49 to 0.36) |  | 0.76 |
| Māori | 0.4 (0.14 to 0.66) |  | 0.**003** |
| Pacific | 0.17 (-0.16 to 0.51) |  | 0.32 |
| **Reflux** |  |  |  |
| Overall effect |  | 0.17 | 0.13 |
| Asian | -0.54 (-1.08 to -0.01) |  | 0.05 |
| Māori | 0.13 (-0.2 to 0.46) |  | 0.43 |
| Pacific | 0.08 (-0.35 to 0.5) |  | 0.73 |

Table 5. Kruskal-Wallis test for associations between GSRS domains and ethnicity at intervention week 0

|  | **P** |
| --- | --- |
| Abdominal pain | **0.03** |
| Constipation | 0.30 |
| Diarrhoea | 0.45 |
| Indigestion | **0.02** |
| Reflux | **0.05** |

Table 6. Correlation and regression coefficients (per unit higher) between GSRS domains and NZ deprivation index

| N=190 | **Spearman’s *r*** | **P** |
| --- | --- | --- |
| Abdominal pain | -0.15 | **0.035** |
| Constipation | -0.02 | 0.73 |
| Diarrhoea | 0.008 | 0.91 |
| Indigestion | 0.01 | 0.86 |
| Reflux | 0.14 | **0.054** |
|  | **NZ Deprivation Change per unit higher (95% CI)** | **P** |
| Abdominal pain | 0.04 (0.001 to 0.08) | **0.04** |
| Constipation | -0.01 (-0.05 to 0.04) | 0.82 |
| Diarrhoea | 0.01 (-0.04 to 0.05) | 0.76 |
| Indigestion | 0.01 (-0.03 to 0.05) | 0.53 |
| Reflux | 0.05 (-0.001 to 0.09) | **0.05** |

Table 7. Correlation and regression coefficients (per decade older) between GSRS domains and age

| N=192 | **Spearman correlation coefficient** | **P** | **Change per decade older (95% CI)** | **P** |
| --- | --- | --- | --- | --- |
| Abdominal pain | -0.10 | 0.18 | -0.10 (-0.20 to 0.01) | 0.06 |
| Constipation | -0.09 | 0.23 | -0.09 (-0.22 to 0.03) | 0.13 |
| Diarrhoea | -0.04 | 0.56 | -0.06 (-0.18 to 0.06) | 0.34 |
| Indigestion | -0.03 | 0.71 | -0.07 (-0.17 to 0.04) | 0.22 |
| Reflux | -0.07 | 0.31 | -0.12 (-0.25 to 0.001) | 0.06 |

Table 8. ANOVA for association between GSRS domains household size

|  | **>2 minus ≤2**  **Estimate (95% CI)** | **R-squared square root (**√R²) | **P** |
| --- | --- | --- | --- |
| Abdominal pain | 0.06 (-0.16 to 0.29) | 0.04 | 0.58 |
| Constipation | -0.12 (-0.39 to .14) | 0.07 | 0.36 |
| Diarrhoea | -0.16 (-0.28 to 0.24) | 0.01 | 0.91 |
| Indigestion | -0.21 (-0.43 to 0.02) | 0.13 | 0.07 |
| Reflux | 0.002 (-0.28 to 0.28) | 0.001 | 0.99 |

Table 9. Mann-Whitney test for associations between GSRS domains household size

|  | **>2 minus ≤2**  **Hodges-Lehmann estimate (95% CI)** | **P** |
| --- | --- | --- |
| Abdominal pain | 0 (0 to 0) | 0.75 |
| Constipation | 0 (0 to 0) | 0.61 |
| Diarrhoea | 0.17 (0 to 0.33) | 0.10 |
| Indigestion | -0.13 (-0.25 to 0) | 0.13 |
| Reflux | 0 (0 to 0) | 0.93 |

Table 10. Correlation and regression coefficients between GSRS domains and PyrMDS total score at intervention week 0

| N=192 | **Spearman correlation coefficient** | **P** | **PyrMDS Change per unit higher (95% CI)** | **P** |
| --- | --- | --- | --- | --- |
| Abdominal pain | 0.006 | 0.94 | -0.04 (-0.11 to 0.04) | 0.34 |
| Constipation | -0.12 | 0.10 | -0.08 (-0.17 to 0.01) | 0.09 |
| Diarrhoea | -0.10 | 0.19 | -0.05 (-0.14 to 0.04) | 0.24 |
| Indigestion | 0.02 | 0.75 | -0.003 (-0.08 to 0.07) | 0.94 |
| Reflux | -0.04 | 0.62 | -0.002 (-0.10 to 0.09) | 0.97 |

**Table 11. Spearman correlation coefficients for associations between 12-week change in PyrMDS total score and GSRS domain scores**

| N=173 | **Spearman correlation coefficient** | **P** | **PyrMDS Change per unit higher (95% CI)** | **P** |
| --- | --- | --- | --- | --- |
| Abdominal pain | 0.002 | 0.98 | 0.004 (-0.07 to 0.07) | 0.92 |
| Constipation | -0.002 | 0.98 | -0.01 (-0.08 to 0.05) | 0.67 |
| Diarrhoea | 0.04 | 0.64 | 0.01 (-0.08 to 0.09) | 0.90 |
| Indigestion | -0.06 | 0.45 | -0.03 (-0.11 to 0.04) | 0.39 |
| Reflux | 0.05 | 0.53 | 0.04 (-0.04 to 0.13) | 0.30 |

Table 12. Spearman correlation coefficients of SF36 dimensions and GSRS component summary scores 12-week change from pre-intervention week 0

| **SF-36 Dimension** | **Abdominal pain** | | **Constipation** | | **Diarrhoea** | | **Indigestion** | | **Reflux** | |
| --- | --- | --- | --- | --- | --- | --- | --- | --- | --- | --- |
|  | **Spearman’s *r*** | **P** | **Spearman’s *r*** | **P** | **Spearman’s *r*** | **P** | **Spearman’s *r*** | **P** | **Spearman’s *r*** | **P** |
| **Bodily pain** | -0.08 | 0.31 | 0.06 | 0.41 | -0.05 | 0.56 | 0.07 | 0.38 | -0.02 | 0.76 |
| **Emotional role** | -0.02 | 0.77 | -0.09 | 0.24 | -0.17 | **0.03** | -0.10 | 0.17 | -0.02 | 0.82 |
| **General health** | -0.25 | **<0.001** | -0.15 | 0.05 | -0.18 | **0.02** | -0.28 | **<0.001** | -0.04 | 0.59 |
| **Mental Component Summary** | -0.13 | 0.09 | -0.21 | **0.006** | -0.18 | **0.02** | -0.17 | **0.02** | 0.12 | 0.12 |
| **Mental health** | -0.10 | 0.20 | -0.16 | **0.04** | -0.07 | 0.34 | -0.13 | 0.09 | 0.08 | 0.33 |
| **Physical Component Summary** | -0.17 | **0.03** | -0.003 | 0.97 | -0.10 | 0.19 | -0.11 | 0.15 | -0.09 | 0.23 |
| **Physical functioning** | -0.10 | 0.21 | -0.11 | 0.14 | -0.14 | 0.07 | -0.16 | **0.04** | -0.05 | 0.49 |
| **Physical role** | -0.05 | 0.52 | -0.05 | 0.51 | -0.09 | 0.30 | -0.05 | 0.53 | -0.14 | 0.06 |
| **Social function** | -0.17 | **0.03** | -0.12 | 0.12 | -0.23 | **0.003** | -0.16 | **0.04** | 0.10 | 0.19 |
| **Vitality** | -0.19 | **0.02** | -0.13 | 0.08 | -0.20 | **0.01** | -0.18 | **0.02** | 0.09 | 0.27 |

Table 13. Regression coefficients displaying the change in SF36 dimension per unit increase in individual GSRS components

| **SF-36 Dimension** | **Abdominal pain** | | **Constipation** | | **Diarrhoea** | | **Indigestion** | | **Reflux** | |
| --- | --- | --- | --- | --- | --- | --- | --- | --- | --- | --- |
|  | **B - Coefficient (CI)** | **P** | **B - Coefficient (CI)** | **P** | **B - Coefficient (CI)** | **P** | **B - Coefficient (CI)** | **P** | **B - Coefficient (CI)** | **P** |
| **Bodily pain** | -1.80 (-3.72 – 0.13) | 0.07 | 0.94 (-1.06 – 2.94) | 0.36 | -0.35 (-1.92 – 1.23) | 0.67 | -0.81 (-2.6 – 0.98) | 0.37 | -0.14 (-1.69 – 1.40) | 0.86 |
| **Emotional role** | -0.60 (-2.73 – 1.52) | 0.58 | -2.09 (-4.26 – 0.09) | 0.06 | -1.80 (-3.5 –  -0.10) | **0.04** | -1.46 (-3.4 – 0.49) | 0.14 | 0.34 (-1.35 – 2.03) | 0.69 |
| **General health** | -3.05 (-4.64 –  -1.45) | **<0.001** | -1.88 (-3.57 –  -0.18) | **0.03** | -1.30 (-2.64 – 0.03) | 0.06 | -2.68 (-4.15 –  -1.20) | **0.001** | -0.07 (-1.39 – 1.25) | 0.92 |
| **Mental Component Summary** | -2.02 (-4.02 –  -0.02) | 0.05 | -2.83 (-4.88 –  -0.79) | **0.01** | -1.6 (-3.22 – 0.02) | 0.05 | -1.98 (-3.82 –  -0.14) | **0.03** | 1.10 (-0.49 – 2.70) | 0.17 |
| **Mental health** | -1.83 (-3.71 – 0.05) | 0.06 | -2.34 (-4.27 –  -0.41) | **0.02** | -0.34 (-1.88 – 1.20) | 0.67 | -1.35 (-3.09 – 0.39) | 0.13 | 0.79 (-0.72 – 2.29) | 0.30 |
| **Physical Component Summary** | -1.27 (-2.75 – 0.20) | 0.09 | 0.01 (-1.53 – 1.54) | 0.99 | -0.67 (-1.87 – 0.53) | 0.27 | -0.76 (-2.12 – 0.61) | 0.27 | -0.59 (-1.77 – 0.59) | 0.32 |
| **Physical functioning** | -0.48 (-2.02 – 1.05) | 0.54 | -1.16 (-2.74 – 0.42) | 0.15 | -0.79 (-2.03 – 0.44) | 0.21 | -0.62 (-2.03 – 0.79) | 0.39 | -0.21 (-1.43 – 1.01) | 0.74 |
| **Physical role** | -0.60 (-2.54 – 1.33) | 0.54 | -0.62 (-2.62 – 1.38) | 0.54 | -0.83 (-2.40 – 0.74) | 0.30 | -0.32 (-2.11 – 1.47) | 0.72 | -0.63 (-2.17 – 0.91) | 0.42 |
| **Social function** | -2.75 (-4.85 –  -0.64) | **0.01** | -2.45 (-4.63 –  -0.27) | **0.03** | -2.37 (-4.07 –  -0.67) | **0.01** | -2.06 (-4.01 –  -0.11) | **0.04** | 0.80 (-0.90 – 2.50) | 0.36 |
| **Vitality** | -2.28 (-4.22 –  -0.35) | **0.02** | -1.76 (-3.77 – 0.25) | 0.09 | -1.51 (-3.09 – 0.06) | 0.06 | -1.88 (-3.67 –  -0.09) | **0.04** | 0.94 (-0.62 – 2.49) | 0.24 |

B-coefficients represent the change in SF-36 dimension per unit increase in GSRS domain score
P-values are equivalent to those derived from Pearson’s product-moment correlation coefficient

B-coefficients represent the change in SF-36 dimension per unit increase in GSRS domain score.
P-values are equivalent to those derived from Pearson’s product-moment correlation coefficient
